# Supplementary material for: Astrocytes modulate brainstem respiratory rhythm-generating circuits and determine exercise capacity
Source: Nat Commun. 2018 Jan 25;9:370. doi: 10.1038/s41467-017-02723-6 (PMC5785528; doi:10.1038/s41467-017-02723-6)
Supplement: Supplementary file 1 — Supplementary Information [file 41467_2017_2723_MOESM1_ESM.pdf]

Supplementary Information

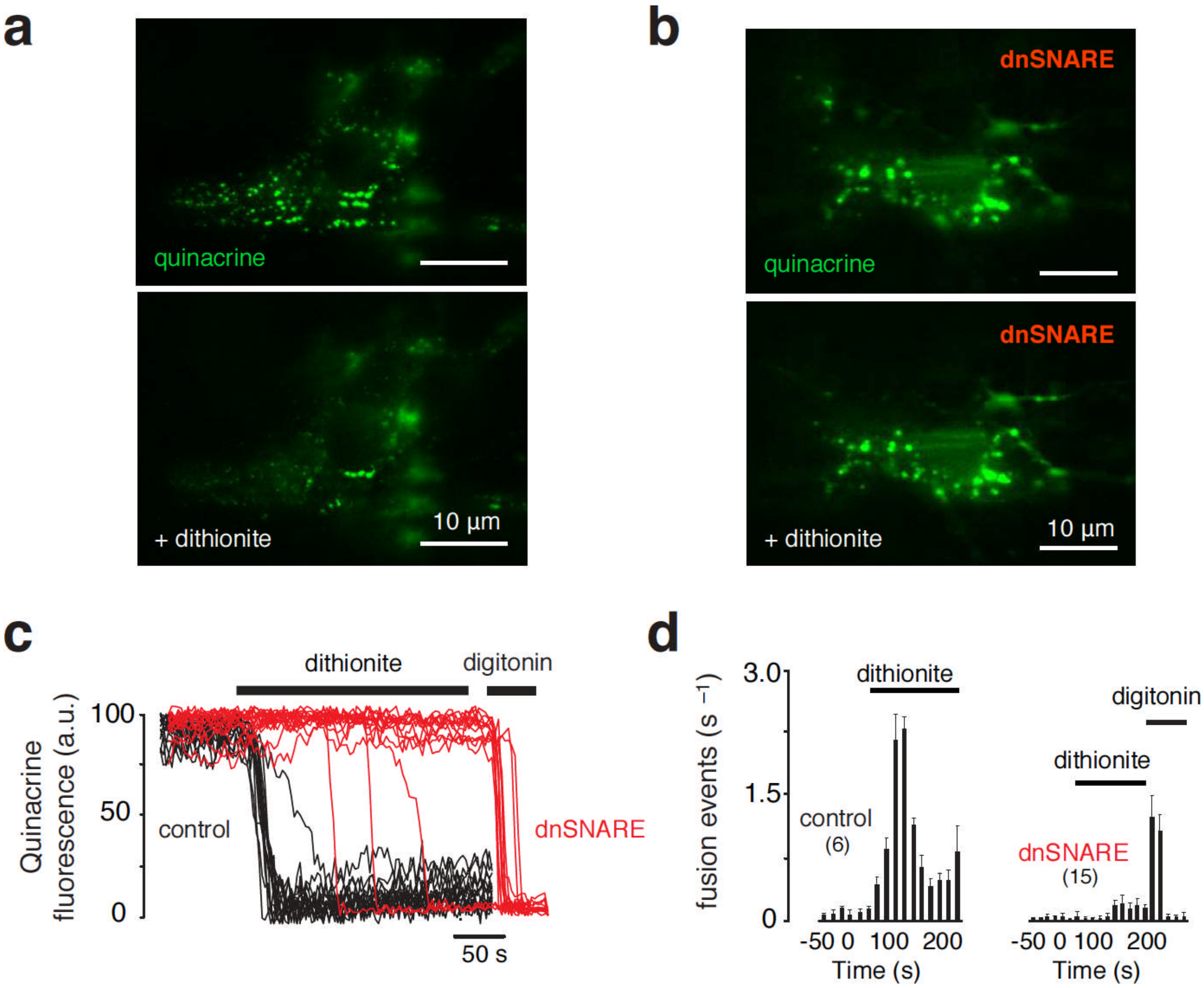

Supplementary Figure 1| Validation of AVV-sGFAP-dnSNARE-EGFP efficacy in blocking hypoxia-induced facilitated vesicular fusion in astrocytes. (**a**,**b**) Total internal reflection fluorescence (TIRF) microscopy images of quinacrine-labeled vesicular compartments in cultured brainstem astrocytes transduced to express control transgene or dnSNARE at resting conditions and after bath application of the oxygen scavenger sodium dithionite. (**c**) Plots of TIRF intensity changes showing loss of quinacrine fluorescence from a proportion of labeled organelles in response to application of sodium dithionite in two individual cultured astrocytes transduced to express control transgene (black traces) or dnSNARE (red traces). In cultures of astrocytes expressing dnSNARE, digitonin was applied at the end of the recording to permeabilize the membranes, resulting in a rapid loss of quinacrine fluorescence. (**d**) Averaged temporal profile of dithionite-induced vesicular fusion events detected in quinacrine-loaded cultured astrocytes expressing control transgene or dnSNARE. Numbers of individual astrocytes recorded in three different experiments are indicated in parentheses.

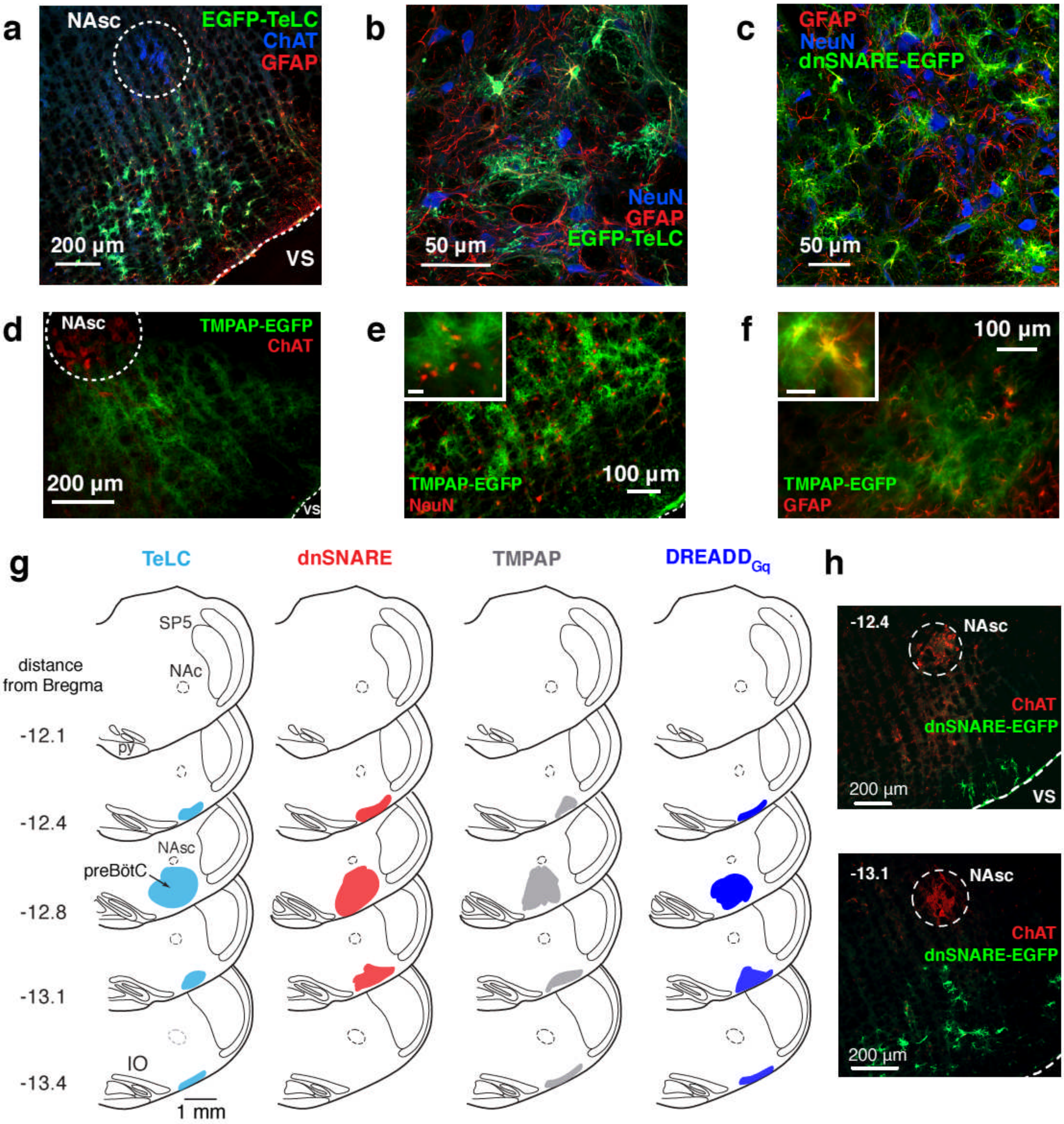

Supplementary Figure 2| Viral targeting of preBötC astrocytes to express TeLC, dnSNARE, TMPAP or DREADD$_{Gq}$. (**a**) Confocal fluorescence microscopy image illustrating EGFP-TeLC expression in a subset of preBötC astrocytes located ventral to the semi-compact division of the nucleus ambiguus (NAsc) identified by ChAT immunoreactivity. (**b**) Representative higher magnification image of preBötC astrocytes targeted to express TeLC. (**c**) Representative confocal image of dnSNARE-EGFP expression in preBötC astrocytes. (**d-f**) Confocal fluorescence images of TMPAP-EGFP expression in the preBötC. Despite the use of the generic promoter (Ef1α), EGFP expression driven by this vector was almost exclusively confined to astroglia (see higher magnification insets in **e & f**; Insert scale bars = 25 µm). VS, ventral surface of the brainstem. (**g**) Schematic illustration of the spatial extent of TeLC, dnSNARE, TMPAP and DREADD$_{Gq}$ expression following viral targeting of preBötC astrocytes in representative brainstems of adult rats from each of the experimental groups. Adenoviral vectors designed to drive the expression of EGFP-TeLC, dnSNARE-EGFP, or DREADD$_{Gq}$-EGFP under the control of the enhanced GFAP promoter were used to

target preBötC astrocytes bilaterally. A lentiviral vector was used to express TMPAP-EGFP under the control of Ef1α promoter. The extent of each transgene expression in the brainstems of all the experimental animals was histologically reconstructed (colored regions shown) from serial coronal sections following GFAP immunostaining. The peak density of transduced astrocytes expressing EGFP was within the preBötC region (extending rostro-caudally from Bregma -12.4 to -13.1 mm), with limited expression in the immediately adjacent rostral and caudal areas of the ventrolateral medullary reticular formation. The preBötC is located ventrally to the NAsc which was identified by ChAT immunostaining of NA motoneurons (see **a**, **d**, **h**) to confirm that the sites of microinjections were centered at the preBötC. No major differences in preBötC expression pattern were found between rats injected with different vectors. In rats transduced to express TeLC, $DREADD_{Gq}$, or CatCh (control), no neuronal expression of transgenes was observed (assessed by NeuN immunostaining, see **b**). In 2 rats transduced to express dnSNARE in the preBötC, ~2% of all the cells expressing the transgene were identified as neurons. IO, inferior olives. NAc, compact division of the NA. py, pyramids. SP5, spinal trigeminal nucleus. **h**) Representative confocal images illustrating dnSNARE-EGFP expression immediately rostral to the preBötC (-12.4 mm from Bregma) and at the caudal boundary of the preBötC (-13.1 mm from Bregma). Main **Fig. 1f** illustrates dnSNARE expression in the preBötC astrocytes corresponding to the brainstem section -12.8 mm (from Bregma).

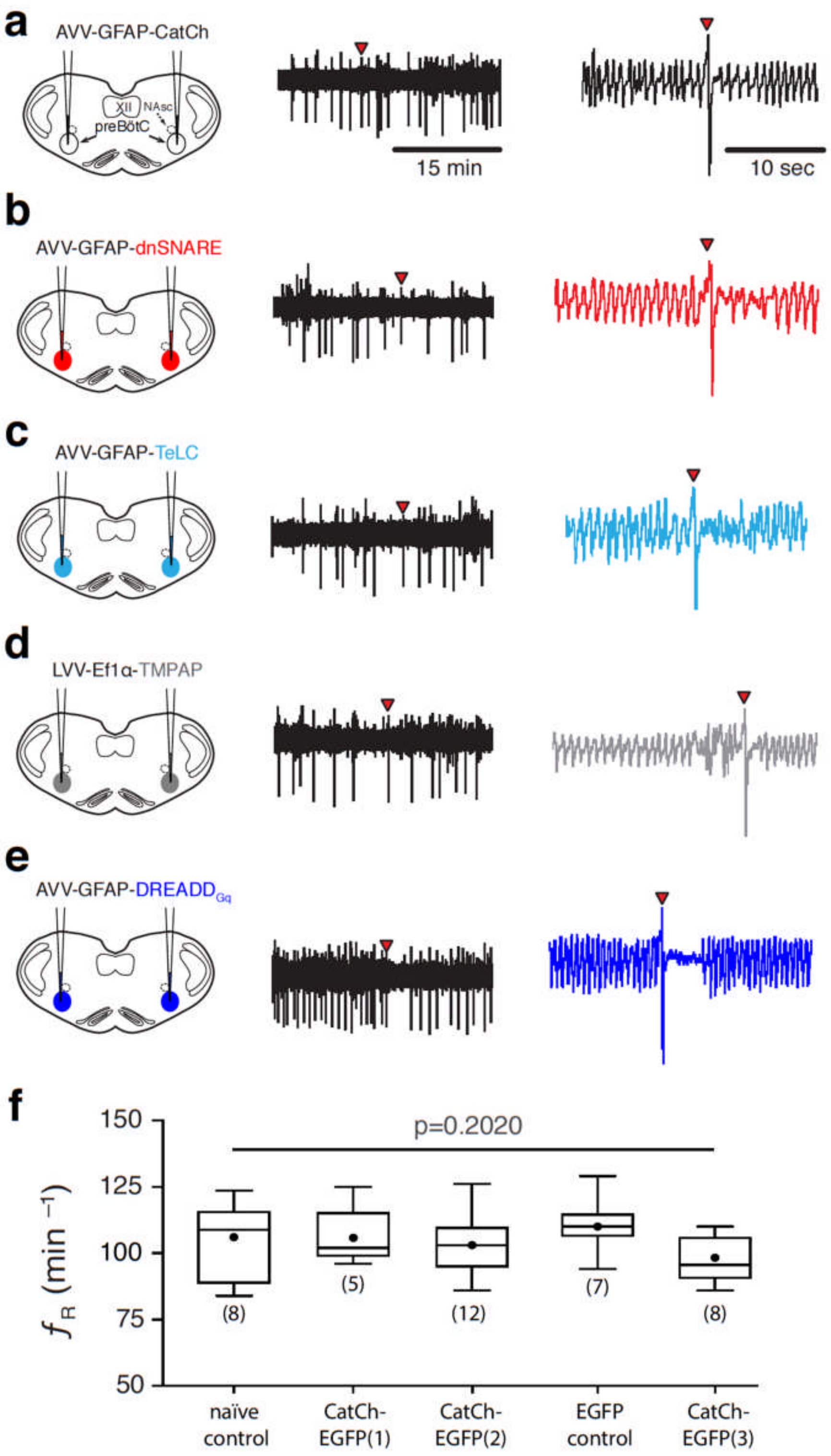

Supplementary Figure 3| Representative whole body plethysmography recordings obtained in conscious adult rats transduced to express CatCh (**a**), dnSNARE (**b**), TeLC (**c**), TMPAP (**d**) or DREADD$_{Gq}$ (**e**) by the preBötC astrocytes. Large amplitude changes in plethysmography chamber pressure [denoted by the arrowheads on the compressed (left) and expanded (right) time scale traces] are indicative of sighs that occur periodically during normal breathing. (**f**) No differences in resting respiratory activity were observed in naïve (non-transduced) rats and rats transduced to express CatCh-EGFP or EGFP in the preBötC. Three different groups of rats transduced to express control transgene CatCh-EGFP [CatCh-EGFP(1), CatCh-EGFP(2), and CatCh-EGFP(3)] were used as time-matched controls for the groups of animals transduced to express dnSNARE, TeLC, and DREADD$_{Gq}$, respectively (for details see section *Methods: Control transgenes*). Rats transduced with a lentiviral vector to express EGFP were used as controls for the experimental animals expressing TMPAP in the preBötC. Numbers of animals for each experimental group are indicated in parentheses. *P* values - Kruskal-Wallis ANOVA by ranks followed by Dunn's *post hoc* test.

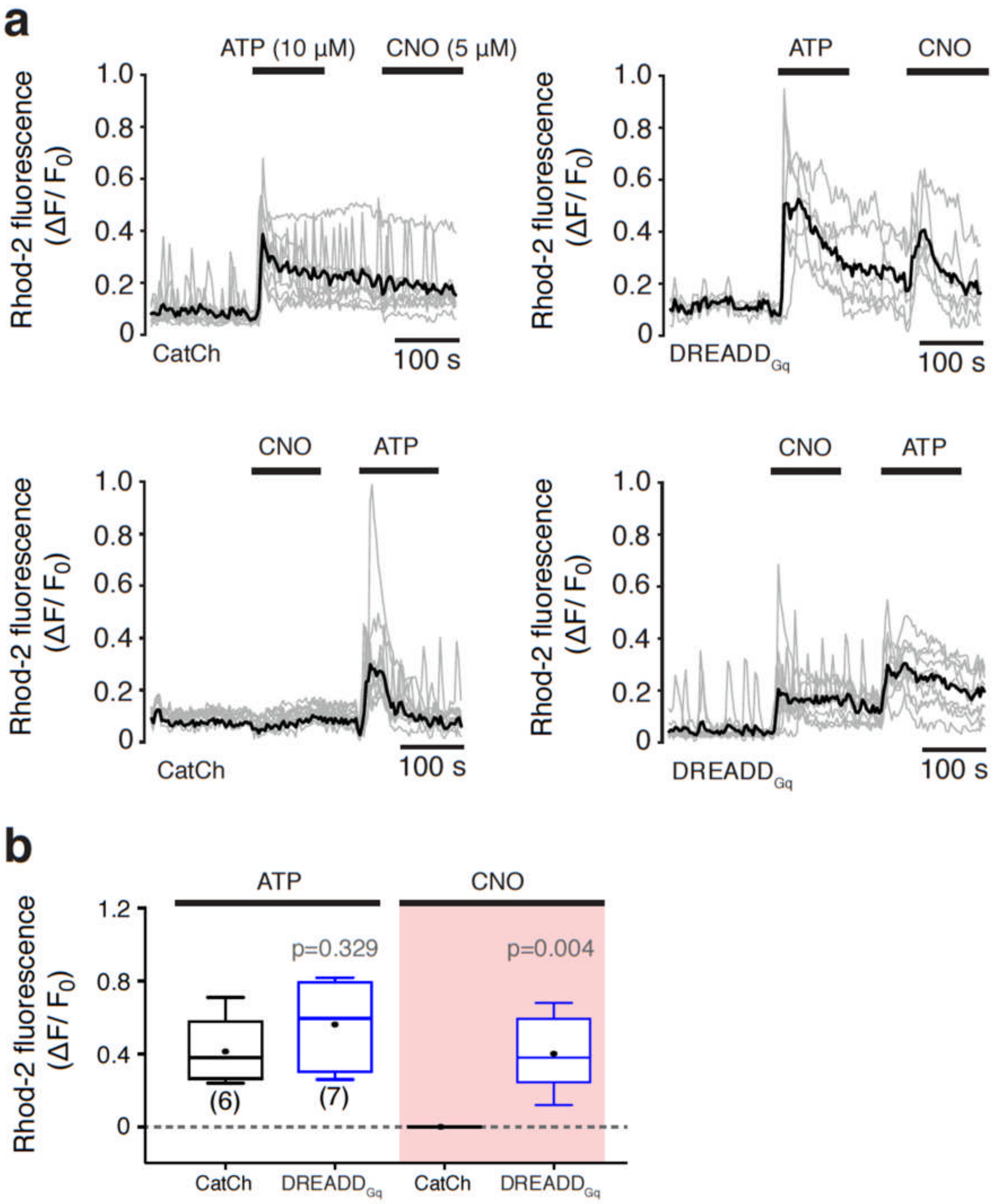

Supplementary Figure 4| Expression of $DREADD_{Gq}$ does not affect the ability of astrocytes to recruit Ca^{2+} signaling pathways triggered by activation of purinoceptors. (**a**) Representative individual (gray) and averaged (black) traces illustrating $[Ca^{2+}]_i$ changes in cultured brainstem astrocytes transduced to express CatCh or $DREADD_{Gq}$ in response to successive applications of ATP and CNO or CNO followed by ATP. (**b**) Summary data illustrating relative amplitudes of ATP and CNO-induced $[Ca^{2+}]_i$ responses in cultured brainstem astrocytes transduced to express CatCh or $DREADD_{Gq}$. Numbers of individual experiments in different cultures are indicated in parentheses. *P* values - Mann-Whitney U rank test.

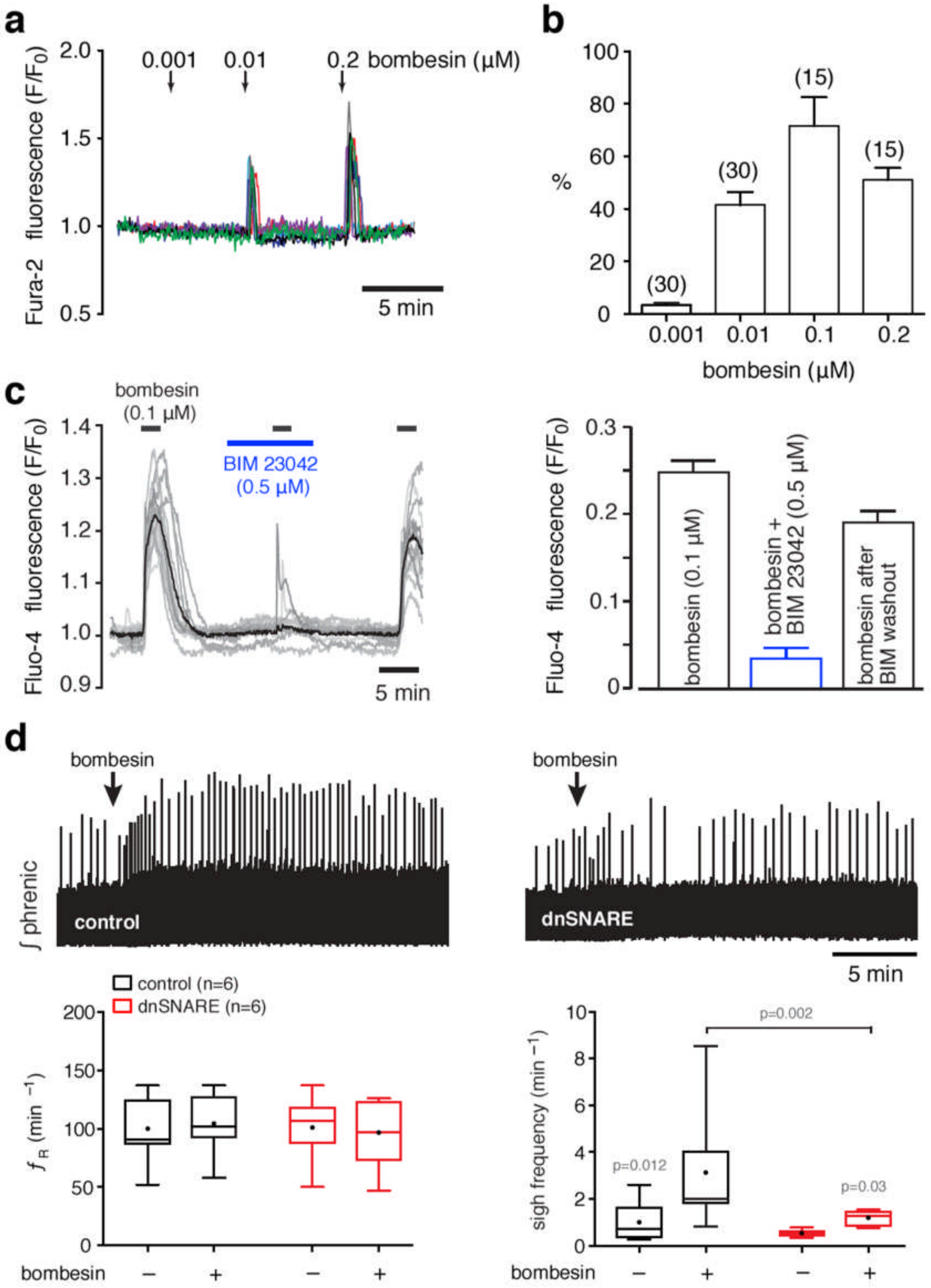

Supplementary Figure 5| PreBötC astrocytes may mediate the effect of bombesin receptor activation on sigh frequency. (**a**) Representative traces illustrating the effect of bombesin on $[Ca^{2+}]_i$ in five individual brainstem astrocytes in culture (traces superimposed). (**b**) Summary data illustrating the percentage of cultured brainstem astrocytes responding to bombesin with elevations in $[Ca^{2+}]_i$. Numbers of individual astrocytes recorded in three separate experiments are indicated in parentheses. (**c**) Representative individual (gray) and averaged (black) traces and summary data illustrating the effect of bombesin on $[Ca^{2+}]_i$ in cultured astrocytes in the absence and presence of selective neuromedin B receptor antagonist BIM 23042. Data in **b**, **c** are presented as means ± SEM. (**d**) Representative recordings of phrenic nerve activity and summary data illustrating the effect of bombesin (250 μM, 50 nl) on resting respiratory rate and sigh frequency following microinjections into the preBötC where astrocytes were transduced to express CatCh or dnSNARE (anaesthetized spontaneously breathing rats). Blockade of vesicular release mechanisms in preBötC astrocytes (dnSNARE expression) significantly attenuated the effect of bombesin on sigh frequency. *P* values - Mann-Whitney U rank test. Data sets with no *p* values indicated are not significantly different.

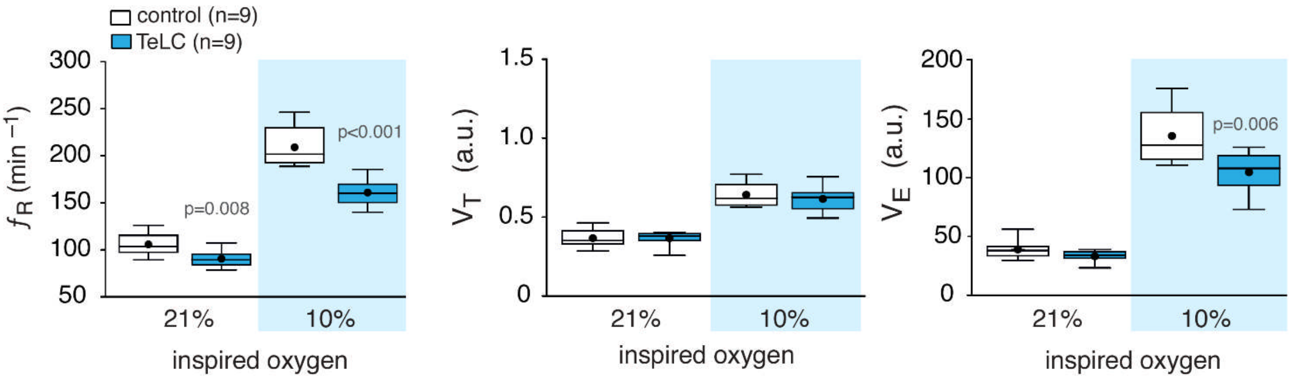

Supplementary Figure 6| Astrocyte signaling within the preBötC contributes to the development of the respiratory response to systemic hypoxia. Summary data illustrating the effects of TeLC expression in preBötC astrocytes on hypoxia-induced increases in the respiratory frequency (f_R), tidal volume (V_T), and minute ventilation ($V_E = f_R \times V_T$) in conscious rats. *P* values - Mann-Whitney U rank test. Data sets with no *p* values indicated are not significantly different.

Supplementary Table 1 | Viral vectors used in this study.

Abbreviated name	Full virus name	Group size	Type	Titer	Target cells
**TeLC**	AVV-sGFAP-EGFP-skip-TeLC	n = 12	adenovirus	$2.1x10^8$	astrocytes
**dnSNARE**	AVV-sGFAP-dnSNARE-EGFP	n = 5	adenovirus	$7.7x10^9$	astrocytes
**$DREADD_{Gq}$**	AVV-sGFAP-$DREADD_{Gq}$-EGFP	n = 8	adenovirus	$8.6x10^9$	astrocytes
**CatCh (control)**	AVV-sGFAP-CatCh-EGFP	n = 5-12	adenovirus	$2.1x10^9$	astrocytes
**TMPAP**	LVV-Ef1α-TMPAP-EGFP	n = 7	lentivirus	$1x10^{10}$	all cells
**EGFP (control)**	LVV-Ef1α-EGFP	n = 7	lentivirus	$1x10^{10}$	all cells
